# Taking Cold

**Published:** 1882-02

**Authors:** 


### TAKING COLD.

KEEP the feet dry and warm and the whole body comfortably clad, and you may bid defiance to all sorts of weather.

The worst colds—those that are followed by dangerous diseases—often come from getting the feet wet and then neglecting too long to change the stockings and shoes for dry ones ; thus the blood becomes chilled and the circulation impeded, so that the waste matters, the products of combustion—as ashes are the product of combustion of fuel—are not carried out of the system, but some remain and become the seeds of disease. Persons who live much of the time indoors suffer more from colds than those who are more exposed to the open air, with all its changes of temperature. If they remain for a few moments in a cold room, or sit near a door or window where they are exposed to a draught of air, they immediately take cold. This often occurs in the fall or early winter, and before recovery from the first attack another follows, so that some persons are hardly free from colds during the winter ; thus the air passages and lungs are all that time in a congested state, which favors the development of diseases peculiar to those organs. There is, fortunately, a remedy against these evils ; and at some period in the future, as remote, perhaps, as the "glacial period" in the past, it may be generally adopted. This remedy is regular exercise or labor in the open air. It is a specific against colds, and, indeed, against most other diseases. No curable case of lung disease can long resist this treatment. To an invalid who can afford it, a saddle-horse is a luxury that makes a pleasure of duty ; but if the finances as well as the lungs are weak, do not be discouraged, but, in Californian parlance, "rough it," and you may recover and enjoy your horse and your fortune afterward.

---

THIS is the way an obituary notice in a Madras paper starts off : "The not uncommon hand of death has distilled with febrile wings from among the débris of bereaved relatives, friends and submissive subjects, into the interminable azure of the past an unexceptionally finished politician and philanthropist of the highest specific gravity," etc. "Débris of bereaved relatives" is good. The writer could make eleven dollars a week in Chicago writing up fires, police reports and divorce cases.—*Norristown Herald.*

